# *Heart health whispering*: A randomized, controlled pilot study to promote nursing student perspective-taking on carers’ health risk behaviors

**DOI:** 10.1186/s12912-018-0291-1

**Published:** 2018-05-24

**Authors:** Michelle Lobchuk, Lisa Hoplock, Gayle Halas, Christina West, Cheryl Dika, Wilma Schroeder, Terri Ashcroft, Kathleen Chambers Clouston, Jocelyne Lemoine

**Affiliations:** 1 Rady Faculty of Health Sciences, College of Nursing, University of Manitoba, Room 315 – 89 Curry Place, Winnipeg, MB R3T 2N2 Canada; 2 Max Rady Faculty of Health Sciences, College of Medicine, University of Manitoba, P228-770, Bannatyne Avenue, Winnipeg, MB R3E 0W3 Canada; 3 Red River College, Nursing, 2055 Notre Dame Avenue, Winnipeg, MB R3H 0J9 Canada; 4 Department of Surgery, Section of General Surgery, University of Manitoba, 770 Bannatyne Avenue, Winnipeg, MB R3E 0W3 Canada

**Keywords:** Education, Empathy, Video-feedback, Health risk behavior, Carers, Nursing students

## Abstract

**Background:**

Lifestyle counseling is described as a “major breakthrough” in the control of chronic diseases. Counseling can be challenging to nurses due their lack of motivation to counsel, hesitancy to appear non-judgmental, lack of empathy, and lack of time. Nurses voice their need for more training in counseling communication skills. Our main objective was to engage in ongoing development and testing of a promising *Heart Health Whispering* perspective-taking intervention on nursing students’ clinical empathy, perceptual understanding, and client readiness to alter health risk behaviors.

**Methods:**

In this randomized controlled pilot study, the full intervention (perspective-taking instructions, practice, and video-feedback) and partial intervention (video-feedback only) comprised 24 and 18 nursing students, respectively. Quantitative data were collected with a 10-item pre- and post-intervention clinical empathy tool, a one-item ‘readiness to change’ health risk behavior tool plus similarity ratings on students’ empathic accuracy were calculated. Data were analyzed using Independent Samples t Tests and mixed model ANCOVA models. Students’ and actors’ evaluative responses toward the intervention phases were collected by handwritten notes, and analyzed using content analysis and constant comparison techniques.

**Results:**

The main finding was that students in the full intervention group reported greater clinical empathy in the post versus baseline condition. Students underestimated their clinical empathy in comparison to carers’ reports in the post-condition. In both intervention groups, carers reported more readiness to change in the post-condition. Carers identified favorable and unfavorable *perceptions and outcomes of approaches taken by students*. Students desired immediate and direct feedback after the video-dialogue and -tagging exercise.

**Conclusions:**

*Heart Health Whispering* is a promising intervention to help educators in basic and continuing education to bolster nurse confidence in empathic conversations on health risk behaviors. This intervention incorporates commonly used strategies to teach empathic communication along with a novel video-analysis application of a perspective-taking task. Student and carer actor comments highlighted the value in opportunities for students to engage in self-evaluation and practicing the empathic process of taking the client’s perspective on health risk behaviors.

**Electronic supplementary material:**

The online version of this article (10.1186/s12912-018-0291-1) contains supplementary material, which is available to authorized users.

## Background

Through the wondrous advancement of medical procedures, treatments and therapies, individuals worldwide are living longer but often with chronic illness [1]. Chronic illness can be costly to affected individuals and to health care systems (i.e., trillions of dollars, globally) [1]. To avert this cost and sub-optimal quality of life often associated with chronic illness, health promotion and disease prevention are “major breakthroughs” in the control of chronic diseases [2]. Evidence demonstrates support for upstream approaches that alter determinants of health by promoting wellness and preventing illness through health behavior choices (e.g., smoking cessation, increased physical activity, and healthy dietary choices) [3–5]. While it remains easier to prescribe medications to avert risk for chronic illness (e.g., aspirin for “cardioprotection”), clinicians’ efforts to promote changes in health risk behaviors are associated with improved mortality rates [6]. However, multiple challenges exist in altering health risk behavior choices.

Health risk behaviors are often longstanding, comforting, and tied to stressful situations, psychosocial variables, work and home environments, limited education, or economic factors [6]. For instance, family carers (aka carers) are a segment of our population that tends to engage in health risk behaviors (e.g., smoking, poor diet, or lack of exercise). This may be due to expectations and stressors associated with the dual demands of caregiving and employment [7, 8]. Consequently, carers may develop mental health issues (e.g., anxiety) and exhibit exaggerated cardiovascular responses to stressful conditions that put them at greater risk than non-carers for the development of high blood pressure or heart disease [9–12].

Despite nurses’ health promotion and illness prevention education, carers consistently report that interpersonal connection and concern for their wellbeing are absent [13, 14]. Nurses have described a lack of motivation to counsel due to the resistance of clients, hesitancy to appear judgmental, lack of empathy due to a poor understanding of individual challenges with behavior change, and struggles in being patient and listening carefully due to the lack of time [15]. However, person-centered approaches require that health care providers endeavor to understand carers and include them as partners in decision-making about behavior change [16]. Our recent pilot work indicated that carer readiness to take ownership for their health risk behaviors is bolstered when nursing students make it a priority to perspective-take, empathically listen, and discern the carer’s unique circumstances, skills, abilities, beliefs, and preferences [17].

Perspective-taking is a teachable component for nurturing clinical empathy. It holds promise in promoting a non-threatening dialogue where individuals can describe their unique contexts and the conditions that underlie unhealthy behaviors or pose barriers to changing unhealthy behaviors [17]. Perspective-taking sensitizes clinicians to: (a) be cognizant of their own thoughts and feelings about health risk behaviors, (b) control their thoughts and feelings to imagine the client’s viewpoint of the health risk behavior and barriers for change, and (c) seek validation of his or her inferences of the client’s viewpoint. Perspective-taking can enhance empathic accuracy (i.e., the ability to accurately infer another person’s thoughts and feelings) [18]. Clients entrust clinicians to take a more person-centered approach and to understand them by identifying obstacles that thwart their readiness to change a health risk behavior [17].

Training opportunities to foster confidence and skills in empathic counseling are needed. This will help nursing students to better understand the underlying influences of health risk behaviors, as well as the stigma and negative attitudes that may implicitly undermine empathic approaches. Our main study objective was to engage in ongoing development and testing of a promising *Heart Health Whispering* intervention as a novel person-centered approach for counseling and health promotion. We posed the following research question: *When nursing students receive perspective-taking instructions and engage in video-feedback (aka Heart Health Whispering)* versus *video-feedback alone, does this result in increased competence in clinical empathy, student-carer perceptual agreement on carer thoughts and feelings about the health risk behavior, and carer readiness to alter health risk behaviors for protection against detrimental cardiovascular conditions?* We also examined evaluative interview responses of students and carers on the impact, appropriateness, and acceptability of the intervention to aid with further refinement.

## Methods

### Study design

We conducted a two-center, randomized, controlled pilot study with a full intervention group (Group I) and a partial intervention group (Group PI). Approvals from the Ethics Boards at the University of Manitoba (aka the university) and Red River College (aka the college) were obtained before executing study protocols. Within the present study, we addressed recommendations from our first one-arm pilot study by: launching a randomized controlled study, incorporating a refined recruitment protocol with nursing students, using carer actors, employing a modified video-tagging session, and obtaining data to calculate effect size differences in students’ clinical empathy [17].

### Participants

Between March 2016 and December 2016, we recruited a convenience sample of undergraduate student nurses and nurse practitioner students. According to our inclusion criteria, the undergraduate sample was comprised of students at: (a) the end of the second year or in the third year of a three-year accelerated baccalaureate program at the college (*n* = 5) or (b) the end of the second year or in the third or fourth year of a four-year baccalaureate program at the university (*n* = 15). All students had completed respective clinical practice, health promotion, relational nursing, family nursing, and adult health courses. Participating undergraduate students received a $20 cash honorarium and a thank you card for their time and effort.

The nurse practitioner sample was comprised of master-level students in a two-year nurse practitioner program at the university who were enrolled in the first term of a clinical practice course (*n* = 22). Students received a pass or fail credit for participating in the study or alternate course assignment. See Additional file 1 for more details on participant recruitment.

No power analysis was performed as this was pilot work addressing participant responses to the recruitment strategy, group assignment, and intervention phases, and the determination of effect size based on the study outcome of clinical empathy for a larger future study.

### Intervention Protocol

**Fig. 1:**
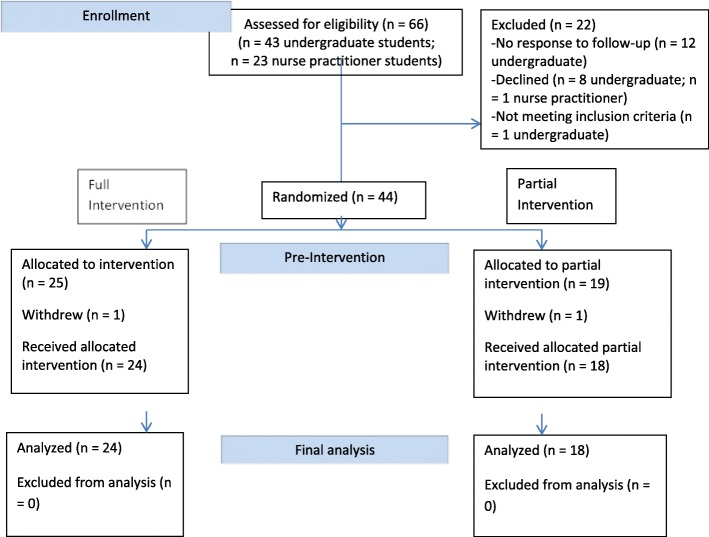
CONSORT DIAGRAM, flow chart of undergraduate nursing students and nurse practitioner students in the full and partial intervention groups

A fulsome description and schematic of the theory-based *Heart Health Whispering* intervention that was influenced by work in social psychology [19–21] has been published elsewhere [17] and is also located in Additional file 2. The full intervention involved four phases. The Research Assistant (RA) conducted a computerized randomization process to assign students to Group I (*n* = 24) or Group PI (*n* = 18) (Fig. 1). Due to practical reasons, students, the interventionist (JL), and interviewers (ML and LH) were not blinded; only the actor was blinded to group assignment. We employed two female actors (hereafter called the ‘carer’) from the College of Medicine, Standardized Patient Program. Actors received a script and training to play the role of a stressed middle-aged family carer who is concerned about her health risk behavior.

*Phase 1* occurred roughly 2 weeks prior to the in-lab session. In this phase, the interventionist taught individual Group I students about perspective-taking and then the students practiced the technique for 2 weeks. Phases 2 to 4 occurred in the lab 2 weeks after Phase 1. At the lab, individual Group I and Group PI students completed demographic and empathy questionnaires. In a separate room from the student, a carer actor used a tool to identify a health risk behavior to discuss with the student and how ready they were to change that health risk behavior. In *Phase 2*, students had a video-recorded dialogue with a carer actor for 10 min about the carer’s health risk behavior. In *Phase 3*, the carer actor was separated from the student. While sitting with the interventionist, the carer watched the dialogue video and paused it each time the carer experienced a thought or feeling. The carer indicated: a) whether it was a thought or feeling, b) whether the thought or feeling was positive or negative in tone, and then c) wrote out in sentence-form the context of the thought or feeling. Later, while the carer was completing Phase 4, the student watched the video with the interventionist. The interventionist paused the video each time the carer had indicated experiencing a thought or feeling. The student then inferred what the carer was experiencing at that time. In *Phase 4*, student and the actor (separately) engaged in exit interviews.

### Data collection and measurement

Students completed the investigator-developed demographic tool to capture student age, gender, extent of communication training with clients, extent of communication training on health risk behaviors, the student’s own experience being a family carer, and the student’s own engagement in health risk behaviors and desire to change said behaviors. The demographic tool took less than 5 min to complete.

We employed an adapted version of the Consultation and Relational Empathy (CARE) [22] tool to capture the student’s inference of the carer’s response to his or her clinical empathy: *Please rate how you feel the carer actor will perceive (perceived) you to be at,* e.g.*,“really listening to him or her”.* Students completed this scale before and after the dialogue which included 10 items; 1 (poor), 5 (excellent); α range = 0.91 to 0.95. The carer completed the original version of the CARE tool [23] after the dialogue to capture her perception of the student’s clinical empathy (α range = 0.94 to 0.95). The CARE tool took less than 5 min to complete.

Before engaging in the dialogue, the carer completed the Risk Factor Identification Tool (RFIT) [24, 25] to identify a health risk behavior to discuss with the student. Because actors posed as the same carer across student encounters, they were able to easily identify a specific health risk behavior for the dialogue. Actors completed the RFIT at the first, middle, and last student encounter in the lab. The RFIT took 10 min to complete.

The Readiness to Change Ruler (aka the Ruler) was used with carers to indicate the health risk behavior that they wanted to discuss with the student and were willing to rate on the Ruler before the dialogue (not ready to change, already changing, and somewhere in the middle; scale ranging from 1 to 10) [26]. To capture differences in the carer’s readiness to change the behavior, the carer was asked to complete the Ruler again after the video-tagging exercise. The Ruler took less than 1 min to complete. The interventionist (JL) shared the pre-dialogue Ruler rating with the student to stimulate dialogue about the health risk behavior.

To measure student perceptual understanding of carers’ thoughts and feelings experienced during the dialogue, we were guided by Ickes’ [21] empathic accuracy approach. We employed scores of “0” (essentially different content), “1” (similar, but not the same), and “2” (essentially the same content) to determine the similarity between student and carer tags involving three evaluative factors of: a) whether the carer experienced a ‘thought or feeling’, b) whether the ‘tone’ of the tagged instance was positive or negative, and c) the ‘situation’ being referenced in the instance [17]. For each tagged instance, rater scores were averaged. The total similarity score was calculated by adding average rater scores for each instance. We then divided the total similarity score by the maximum points that the student could have obtained to obtain an overall percent similarity score. Fleiss Kappa coefficients for agreement among six investigative team raters across ten students’ tagged instances were 0.49 (*p* < 0.0001) indicating moderate rater agreement; for “0” was 0.65 (*p* < 0.0001) indicating substantial agreement; “1” was 0.38 (*p* < 0.0001) or fair agreement; and, “2” was 0.54 (*p* < 0.0001) or moderate agreement [27].

Open-ended exit interviews, as guided by investigator-developed scripts (Tables 1 and 2), were conducted (ML, LH, JL) respectively with each student and carer. Handwritten notes on participant responses about the impact, appropriateness, and acceptability of each phase of the intervention protocol were captured. The exit interviews took 30 min to complete.

**Table 1:** Interview questions for nursing students

Question #1	
Full intervention group	Can you describe your thoughts and feelings about the empathic technique when you applied it with the carer to help you understand his or her thoughts and feelings about health-risk behaviour?
Partial intervention group	Can you describe the approach that you took with the carer to help you understanding his or her thoughts and feelings about the health-risk behavior? Can you describe your thoughts and feelings about the approach you took with the carer to help you understand his or her thoughts and feelings about health-risk behavior?
Question #2	
Full intervention and Partial intervention groups	Can you tell me whether you think that the empathic technique (or approach you took) helped you to better understand the carer’s thoughts and feelings about health-risk behavior? Prompt: If yes, I would be interested in knowing ‘how’ the intervention (or approach you took) helped you? Prompt: Did the intervention (or the approach you took) affect how you felt about the carer? Prompt: Did the intervention (or approach you took) affect your behavior toward the carer? How did the intervention (or approach you took) affect your behavior toward the carer? Can you describe whether your approach in trying to understand health-risk behaviors is different now since you learned this new technique (full intervention group only)? Prompt: Can you tell me whether you plan to continue using this technique (or your approach) to help you understand health-risk behaviors of patients and carers better? If no, why not? If yes, why? Prompt: I am also interested in knowing if the technique (or approach you took) caused you to learn something different about yourself and how you view health-risk behaviors?
Question #3	
Full intervention and Partial intervention groups	As a last question, is there anything else you would like to add to help the researchers of this study develop this intervention to help student nurses talk with carers about health- risk behaviors? Example: Were there any comments or concerns about the appropriateness and clarity of demographic data questions and the video-tagging exercise?

**Table 2:** Interview questions for carer actor

Question #1	Can you describe your thoughts and feelings about the approach the student nurse took to help him or her understand your thoughts and feelings about the health-risk behavior?
Question #2	Can you tell me whether you think that the approach the student nurse took helped him or her to better understand your thoughts and feelings about the health-risk behavior? Prompt: If yes, I would be interested in knowing ‘how’ the student’s approach helped you to describe your thoughts and feelings about the health-risk behavior? Prompt: Did the student nurse’s approach affect how you felt about the student nurse? Prompt: Did the student nurse’s approach affect your behavior toward the student nurse?
Question #3	Can you describe whether your ‘personal’ approach (s) will be different when you talk about your own wellness and health-risk behaviors with other health care providers (e.g., your physician, other nurses)? Prompt: Can you tell me whether you plan to continue using this approach to help your own health care providers better understand your health-risk behaviors? If no, why not? If yes, why?
Question #4	I am also interested in knowing if the student’s approach caused you to learn something different about yourself and how you view health risk behaviors?
Question #5	As a last question, is there anything else you would like to add to help the researchers of this study develop this intervention to help student nurses talk with carers about health- risk behaviors? Example: Were there any comments or concerns about the appropriateness and clarity of study questionnaires (i.e., the computer risk identification tool, the ‘readiness to change’ ruler, and the CARE tool) and the video-tagging exercise?

### Statistical analysis

Analyses were carried out with SAS Software (version 9.4) Institute Inc., Cary, NC, USA. Due to the small size of undergraduate and nurse practitioner sub-groups, data from all participants (*n* = 42) were aggregated for inferential analysis. Twenty-four students were in Group I and 18 were in Group PI. All tests of significance were set at *p* < 0.05.

Descriptive analyses (means, medians, standard deviations, and frequencies) of participant characteristics and study variables were conducted. An Independent Samples t Test examined whether there was a significant difference in *student-carer perceptual agreement* on carer thoughts and feelings about the health risk behavior (i.e., similarity scores) between intervention groups. Mixed model ANCOVA models were used to test for significant differences between intervention groups on their average effects on *student clinical empathy* (CARE tool) and *carer readiness to change* (the Ruler tool) scores: factor 1 = intervention groups; factor 2 = time; interaction = time by intervention group. Independent Samples t Tests tested for significant differences in mean CARE and Ruler scores between groups.

Two authors (ML and LH) independently examined hand-written notes of exit interview responses by integrating field notes and using content analysis and constant comparison techniques to identify, code, categorize, and label the primary patterns in the data [28, 29]. When disagreements occurred in the coded data, discussions occurred between ML and LH until consensus was achieved. Supporting quotes from the data were selected for identified themes and sub-themes.

## Results

A total of 42 nursing students provided voluntary consent to participate. The undergraduate nursing students (*n* = 20) were between 19 and 35 years of age; nurse practitioner students (*n* = 22) were between 25 to 51 years of age. Across all students, the majority (88%) were female who reported having received communication training to engage with clients in undergraduate coursework, continuing education, or job training. Fifty-two percent of students reported having previously received communication training to dialogue with clients on health risk behaviors in courses at the undergraduate and/or graduate levels of study. Seventeen per cent (*n* = 7) of the participants stated that they were a family carer and 57% (*n* = 4 of 7) had care recipients who engaged in health risk behaviors (e.g., poor dietary intake, misuse of alcohol, and lack of exercise). Regarding student participants’ own health risk behaviors, 48% indicated that they had behaviors (often multiple) that they wished they could change (e.g., poor diet, smoking cigarettes or marijuana, lack of exercise, inadequate sleep, and poor coping with stress) (Table 3).

**Table 3:** Descriptive statistics of nursing students (*n* = 42)

Variable	N (%)
Gender	
Female	37 (88%)
Male	5 (12%)
Age, years (mean; range)	
Undergraduates	25.3 (19 to 35)
Nurse Practitioner	33.0 (25 to 51)
Year in nursing program	
Undergraduates	
2nd year (end)	2 (5%)
3rd year	7 (17%)
4th year	11 (26%)
Nurse Practitioners	
1st year	22 (52%)
Nursing school	
Undergraduates	
College	5 (12%)
University	15 (36%)
Nurse Practitioners	
University	22 (52%)
Received communication training with carers?	
Yes	22 (52%)
No	19 (45%)
Missing	1 (3%)
If yes, what type of communicating training?	Specific courses in undergraduate program (*n* = 11) Non-specific courses in undergraduate program (*n* = 7) Home care companion / Health care Aide orientation training (*n* = 1) Continuing education (n = 2)
Received communication training about health risk behaviours?	
Yes	19 (45%)
No	22 (52%)
Missing	1 (3%)
If yes, what type of health risk communication training?	Specific courses in undergraduate/graduate program (*n* = 9) Non-specific courses in undergraduate/graduate program (n = 6)
Are you currently a family carer?	
Yes	7 (17%)
No	35 (83%)
Does your care recipient engage in health risk behaviors?	
Yes	4 (57%)
No	3 (43%)
If yes, what type of health risk behaviors?^a^	Poor dietary intake (*n* = 2) Alcohol, smoking, drug, illicit and prescription drug abuse, sexual exposure, lack of exercise, poor diet (*n* = 1) Self-harm, illicit drugs, and manic behaviours (n = 1)
Do you have any health risk behaviors you wish you could change?	
Yes	20 (48%)
No	19 (45%)
Missing	3 (7%)
If yes, what type of health risk behaviors?^a^	Poor diet (*n* = 4) Smoking (*n* = 1) Poor stress coping and lack of exercise (*n* = 1) Poor diet, secluding self when sad or overwhelmed, cigar and marijuana smoking (*n* = 1) Poor diet, lack of exercise, inadequate sleep, poor stress coping (*n* = 1) Poor diet, lack of exercise, inadequate sleep (*n* = 1) Poor diet, lack of exercise, poor diet, poor stress coping, and smoking (*n* = 1) Not identified (*n* = 6)

^a^Participants identified multiple health-risk behaviors either for their care recipient or themselves

### Quantitative findings

Regarding differences in mean *clinical empathy* (CARE tool) (Table 4), a mixed-model ANCOVA analysis revealed no main effects of intervention group, *F*(1,40) = 0.75, *p* = 0.391. Time was significant, *F*(1,40) = 7.94, *p* = 0.008 as well as the interaction between intervention group and time, *F*(1,40) = 9.98, *p* = 0.003. Within-group analysis revealed that students in Group I reported having more clinical empathy at post-measurement than they did at baseline, *t*(40) = 4.56, *p* < 0.0001; 95% CI [2.16, 5.59]; *d* = 0.43. There was no statistically significant difference between baseline and post-intervention clinical empathy in Group PI; *t*(40) = − 0.23, *p* = 0.822; 95% CI [− 2.20, 1.76]; *d* = 0.04. Between-group analysis revealed no significant difference in clinical empathy between Group I and Group PI at baseline; *t*(40) = − 0.23, *p* = 0.822; 95% CI [− 4.41, 3.52]; *d* = 0.07. There was also no significant difference in clinical empathy between Group I and Group PI, post-intervention; *t*(40) = 1.86, *p* = 0.07; 95% CI [− 0.31, 7.62]; *d* = 0.62. Thus, while Group I and Group PI students scored similarly on clinical empathy, Group I students perceived themselves to increase in empathy, whereas Group PI students did not.

**Table 4:** Differences in CARE scores of student nurses and carers

	Baseline Condition		Post-Intervention Condition	
	Intervention Group (I) (Mean, SD)	Partial Intervention Group (PI) (Mean, SD)	Intervention Group (I) (Mean, SD)	Partial Intervention Group (PI) (Mean, SD)
Students	31.67 (6.88)^a^	32.11 (6.27)	35.54 (6.35)^a,d^	31.89 (5.33)^c^
Caregiver	–	–	40.92 (9.91)^b,d^	46.22 (5.24)^b,c^

Common superscripts indicate the same column or row mean scores were significantly different from each other; ^a^identifies within-group differences; ^b-d^identifies between-group differences. CARE tool range of scores 0 to 50 units (higher ratings indicate greater clinical empathy)

Supplemental analysis revealed a significant difference between Group I students and carers, *t*(39.14) = 2.24, *p* = 0.03; 95% CI [0.52, 10.23]; *d* = 0.65, on student clinical empathy, post-intervention. Similarly, there was a significant difference between Group PI students and carers, *t*(34) = 8.13, *p* < 0.0001; 95% CI [10.75, 17.92]; *d* = 2.71, post-intervention. Thus, both groups of students underestimated their clinical empathy in comparison to carers’ reports. Of interest, carers’ perceptions of student clinical empathy was lower for Group I than for Group PI students; *t*(36.49) = − 2.24, *p* = 0.031; 95% CI [− 10.11, − 0.50]; *d* = 0.67 (Table 4).

With regard to *student-carer perceptual agreement* (i.e., similarity ratings), the overall percent similarity score was higher in Group I (mean 68.41, SD 10.33; range 41.67 to 85%) across 236 instances compared to Group PI (mean 65.24; SD 16.95; range 30.68 to 90%) across 189 instances. However, this difference between the two groups was not statistically significant, *t*(26.28) = 0.70, *p* = 0.489; 95% CI [− 6.11, 12.46]; *d* = 0.23. Overall, there was tendency for students in Group I to achieve greater perceptual agreement on carers’ thoughts and feelings about health risk behaviors than students in Group PI.

On *carer readiness to change* (the Ruler tool), a mixed-model ANCOVA revealed no main effects of intervention group (*F*(1,40) = 0.00, *p* = 0.974) or interaction effects (*F*(1,40) = 0.00, *p* = 0.998). Time was significant, *F*(1,40) = 82.10, *p* < 0.0001. Within-group analysis revealed that carers in Group I reported more readiness to change at post-measurement (mean 8.22, SD 1.50) than they did at baseline (mean 5.91, SD 1.84), *t*(40) = 6.92, *p* < 0.0001; 95% CI [1.64, 2.99]; *d* = 1.38. Similarly, Group PI carers reported more readiness to change at post-measurement (mean 8.21, SD 1.65) than they did at baseline (mean 5.89, SD 1.96); *t*(40) = 6.00, *p* < 0.0001; 95% CI [1.53, 3.09]; *d* = 1.28. Thus, carers reported a similar readiness to change post measurement regardless of whether they interacted with students in Group I or Group PI.

### Evaluative interview responses

Evaluative responses by carers toward students’ approach (Additional file 3) and students’ responses toward intervention phases (Additional file 4) are displayed along with descriptive response counts and illustrative quotations for each sub-theme. A cursory examination revealed a similar count across most sub-themes by carers and students in both intervention groups. The following summary focuses on the most frequent comments in both groups, except when noted.

#### Carers

Carers identified varied favorable and unfavorable *perceptions and outcomes of behavior or approaches* taken by students in the dialogue. Favorable comments included ‘good communication and listening skills’ that were congruent with ‘nonverbal communication skills’ and evaluated by carers as exhibitions of ‘empathy’ and caring or intuitive ‘responsiveness’. Carers appreciated students who employed a ‘gentle, educational approach’ about managing the behavior. Although carers felt that Group I students provided more ‘summarization’ of the dialogue, carers in both intervention groups ‘felt understood’ and ‘felt safe and comfortable in not being judged’. When interacting with Group I students, carers ‘felt new resolve to change’ their health risk behaviors. They described that they ‘learned to take the initiative’ in the future to openly share authentic information with their own health care providers. Carers in both intervention groups said that they ‘learned more about [their] own feelings’ of resentment and guilt relating to their caregiving situation. When carers described ‘liking the student or their approach’, they felt a connection with the student, which occurred particularly when students’ asked the right question and focused on the carer’s self-care.

Unfavorable comments by carers included descriptions of when ‘students needed guidance’ from the carer who had to take the lead in the dialogue. For example, during one session, the carer liked that the student allowed the carer to take the lead in the dialogue, however the carer also felt compelled to start the conversation due to student reticence: “She had a very caring, validating, non-judgmental approach. But she didn’t explore in any great depth [my] motives for binge-eating. I felt that she was reticent to explore further or didn’t feel comfortable delving deeper” (D3 UG Group PI). Of note, carers described ‘not feeling heard’ by some students in Group I. A number of Group I students tended to take ‘paternalistic’ stances by offering inappropriate advice or making assumptions about the carer. Carers described ‘no delving and/or jumping to intervene’ for some Group I students who seemed eager to intervene before fully understanding the carer’s triggers for engaging in the health risk behavior. There were additional carer comments about some Group I students who were described as ‘inauthentic’ in their approach based on behavioral signals (limited eye contact) or robotic verbal responses. Overall, carer actors described a range of mixed emotions and experiences with students in both groups.

#### Students

In Phase One, students practiced perspective-taking with close others (e.g., their parents and friends) who engaged in health risk behaviors such as smoking and poor diet. Most comments reflected how the practice session helped students to attain a ‘better comprehension of others in terms of triggers for engaging in the health risk behavior. A number of students indicated that engagement in perspective-taking was ‘not easy to do’. Perspective-taking required a different approach by students. In particular, it required more conscious effort and students reported that they needed to practice taking the other person’s viewpoint rather than resorting to a familiar self-oriented way of thinking or making assumptions about another person’s risk-taking behavior.

For Phase Two, students revealed three goals for the dialogue: build trust and respect, seek understanding, and provide support. To fulfill these goals, students identified helpful verbal and non-verbal ‘communication techniques’, such as paraphrasing and maintaining an open body posture. As a result of these techniques, which included perspective-taking (mentioned more frequently by students in Group I), both groups ‘gained an appreciation of the carer’s perspective’ on their health risk behavior as a coping technique. One Group I student stated that the intervention helped her to see that there was more to the carer’s situation than just smoking. She thought the dialogue would have been unsuccessful if she had tried to intervene instead of listening to the carer. Despite our instructions to not intervene and seek understanding, students mentioned that they had ‘wanted to intervene’ and provide the carer with suggestions for improving their situation. Group PI students commented more frequently about their ‘self-reflection on improvement’ of communication techniques that would come with more practice. Students in both intervention groups perceived Phase Two to be ‘a positive experience’ with greater ‘self-awareness’ (of non-verbal cues). They recommended that video-recorded dialogue be incorporated into the curriculum. One student exclaimed, “We need to practice these skills, like today!” (D8 UG Group PI).

Students in both intervention groups commented on their ‘appreciation’ for Phase Three as an opportunity to see themselves in a new light and think about different approaches they might adopt in similar situations in practice. Reviewing the dialogue video and engaging in video-tagging appeared to be particularly helpful for students to engage in ‘self-reflection on improvement’. One student reported, “I think the video-tagging process is a really great way to pick up on nuances of therapeutic communication, especially non-verbal communication. I think it is a really great way for students to fine tune communication skills in a non-threatening environment so that they may be applied in clinical practice” (D13 UG Group I).

The field notes revealed recommendations for improvement in student video-feedback. Students desired immediate and direct feedback from the carer after the dialogue and/or after completing the tagging: e.g., how accurate they were in the tagging. Although students received this feedback after data analysis, they preferred to receive it more immediately. Taken together, results indicate that students perceived their participation to be worthwhile and the protocol to be effective, appropriate, and acceptable.

## Discussion

Family carers need to take care of their wellbeing, but this often does not happen. The ability of nurses to counsel carers on their health risk behaviors in order to maintain or achieve wellness relies on an empathic, therapeutic approach and building relationships with them [30]. Developing the capacity of nurses to demonstrate clinical empathy is “best achieved at the student level” while their attitudes and skills are being formed [31, 32]. In the present study, we followed up on our one-arm pilot study [17] by conducting a two-arm randomized control trial. We sought to obtain preliminary data on the immediate impact of the *Heart Health Whispering* intervention on: student competence in clinical empathy, student-carer perceptual agreement on carer thoughts and feelings about their health risk behaviors, and carer readiness to change the health risk behavior.

The main finding in this study is that students in the full intervention group (Group I) reported greater clinical empathy after engaging in perspective-taking training (post versus baseline condition). Students in the partial intervention group (Group PI; who did not receive perspective-taking instructions or the opportunity to practice perspective-taking) reported no difference in their clinical empathy. These findings support recommendations to include theoretical, didactic, and experiential learning to bolster student knowledge of empathy and the underlying process of perspective-taking [33–35]. Students’ qualitative comments demonstrated that their customary responses toward health risk behaviors are driven by erroneous assumptions of individual choice rather than seeking to understand individual circumstances and perspectives regarding the difficulties in changing problematic behaviors. Their comments corroborated previous findings on the value of perspective-taking in allowing them to enhance their ‘exploring skills’ [36] and encouraging carers to gain trust in exploring sensitive triggers for health risk behaviors. The present study provides promising evidence that the 20–30 min instructional session and a 2-week practice session had a distinct contribution in enhancing students’ empathic approach. However, larger studies on the ‘dose’ of instructional and practice sessions are required before educators incorporate the perspective-taking exercise into educational curricula.

On a promising note, our cursory analysis indicated that post-intervention clinical empathy and similarity scores were greater for Group I students whose qualitative comments indicated a greater appreciation for the carer’s viewpoint on health risk behaviors than Group PI students. The lack of significant between-group differences in the post-condition in student self-reports on clinical empathy, carer readiness to change, and student-carer similarity ratings can be attributed to: possible contamination (i.e., Group I students came in contact with Group PI students and may have disseminated perspective-taking information shared by the RA), the small sample size, or the research design (i.e., both groups of students received similar benefits of video-feedback such as the opportunity for self-reflection on their communication styles). Exit interview findings, combined with actors’ CARE and readiness to change responses, suggest that students in both groups demonstrated empathy, even if Group PI students did not receive the full intervention. We speculate, as have other researchers [16, 37–40], that having the undivided attention of students who focused on carer self-care helped the carer to take ownership, feel empowered, become positive about personal strengths, and have confidence to change a challenging behavior; this is also supported in a review on the positive impact of self-affirmation on health behavior change [41]. Thus, nursing students were receiving good communication training and acquiring fundamental communication skills. There is potential to enhance this further through the *Heart Health Whispering* intervention introduced in this study.

The second interesting finding is that carers rated student clinical empathy as higher than students’ self-assessment. This finding is consistent with a meta-analysis that revealed a limited linkage between objective measures and self-reports on perceptual accuracy [42]. Clinicians are poor judges of their consultation performance in the context of health risk behavior conversations [43]. Similarly, early researchers in social psychology found that ‘observer-target’ similarity ratings do not associate well with observer self-reports on empathic accuracy [44]. Kalish et al. [45] speculated that their findings of higher preceptor ratings than students’ ratings on student compassion were due to different expectations. Students may have also been unduly critical in their approach [45]. Together, these findings indicate that our continued use of objective measures of individuals’ attempts to engage in empathy-related behaviors and empathy-related outcomes is needed to establish the validity of self-reports.

A third interesting, but unexpected finding is that carers rated clinical empathy as significantly greater for Group PI students versus Group I students, post-condition. A differential count of qualitative responses showed that carers reported they felt ‘less heard’ in Group I compared to Group PI. Carers also commented on students in Group I ‘not delving enough’, and seeming ‘inauthentic’, and ‘paternalistic’ in their responses to the carer. Plausible reasons for this could be that Group I students may have become impatient based on their comments on the difficulties they experienced when engaging in perspective-taking. Jansink et al. [15] described that nurses tend to leap “ahead of the patient” and hold “false or too high expectations” for motivation to change. Perspective-taking requires careful listening skills that are different from customary quick provision of health risk behavior information and advice. On the other hand, Group PI students had less cognitive demands and likely were less distracted in their dialogue allowing them to draw on previously acquired knowledge and skills that was detected by the carer. This is supported by the qualitative responses of Group PI students where they indicated more frequent use of communication techniques than did Group I students. Additionally, Group I students were struggling with practicing their new skills resulting in poorer performance than students in Group PI. Miller and Mount [46] described that learning and practicing new skills takes time and can lead the trainee to experience ill ease and the lack of genuineness. This is consistent with what carers detected in the present study. Carers added that some students required more practice; this is supported by growing evidence on the benefits of repetitive practice and feedback sessions [47, 48] with standardized actors and peers or fellow trainees [37, 49] in tailored clinical contexts where skills will be applied.

The present study has several limitations that pose as potential threats to internal validity (e.g., small scale study, cross-sectional measurement, and contamination). A main concern that requires ongoing attention is the challenge that raters encountered in not achieving substantive agreement on similarity ratings, particularly on the “1” rating (i.e., similar, but not the same content). A key recommendation by the raters, was to develop clearer qualitative decision rules; for each tagged instance, raters should first evaluate ‘the situation’ followed by ‘the tone’, and then the ‘thought’ or ‘feeling’. We also did not conduct observational evaluations of students’ perspective-taking behaviors that are often incorporated in communication training. However, our main aim was to continue to develop and test our empathy-based video-analysis intervention, and determine its impact on students’ perceptual understanding of carers’ thoughts and feelings about health risk behaviors.

Notable strengths to this research include our ongoing systematic development of our theory-derived intervention. We addressed key recommendations from our previous pilot work to improve study protocols, including improved recruitment strategies and enhanced instructions for the video-tagging exercise. We employed a highly reliable clinical empathy tool that demonstrated sensitivity to detect differences in pre- and post-conditions. Although *Heart Health Whispering* is comprised of a combination of new and previously developed strategies, our innovative approach integrated them to enhance the way students learn the perspective-taking approach. Our approach encouraged self-awareness of one’s beliefs on health risk behaviors, provided perspective-taking instructions with practice sessions, provided the opportunity to hear stories about the impact of the carer role and potential linkages with carers’ health risk behaviors, and engaged students in a novel video-analysis application of a perspective-taking task designed to help them infer carers’ thoughts and feelings [33, 50–53]. Evaluative feedback obtained from carers and nursing students allow us to make continued improvements to the intervention (i.e., provide more direct and immediate sharing of tagged video-data with students) and develop a reliable similarity rating tool that captures students’ perceptual understanding.

## Conclusions

While developing and studying the impact of *Heart Health Whispering*, outcomes for students and carers appeared promising and warrant further testing. The video-analysis (tagging video instances) and -feedback (i.e., sharing similarity scores) components of the *Heart Health Whispering* intervention are novel. Nurse educators in basic and continuing education are offered preliminary evidence on how to bolster nurse confidence in having empathic conversations about health risk behaviors. Carers said that empathic conversations help them to take ownership, feel trust that they are understood without judgment, and gives them a voice in exploring solutions as revealed through their stories. Nursing students and practicing nurses who learn these techniques could be encouraged to engage in self-evaluation plus annual re-training or reassessment as part of self-competencies in communication skills. This study has implications for ongoing empirical work to further refine a promising intervention on how to bolster clinician clinical empathy so as to avoid “jumping ahead of the patient” [15] when engaging in therapeutic conversations on carer self-care.

## Additional files

Additional file 1: Recruitment Protocol for Undergraduate and Nurse Practitioner Students. (DOCX 14 kb)

Additional file 2: Intervention Protocol. (DOCX 19 kb)

Additional file 3: Family Carer Actor qualitative table. (DOCX 25 kb)

Additional file 4: Student qualitative table. (DOCX 25 kb)

## Abbreviations

ANCOVA: Analysis of covariance
CARE: Consultation and relational empathy measure
carers: Family carer actors
Group I: Intervention group
Group PI: Partial intervention group
RA: Research assistant
RFIT: Risk factor identification tool
Ruler: Readiness to change ruler
