# Supplementary material for: Heart health whispering: A randomized, controlled pilot study to promote nursing student perspective-taking on carers’ health risk behaviors
Source: BMC Nurs. 2018 May 24;17:21. doi: 10.1186/s12912-018-0291-1 (PMC5968556; doi:10.1186/s12912-018-0291-1)
Supplement: Supplementary file 1 — Recruitment Protocol for Undergraduate and Nurse Practitioner Students. (DOCX 14 kb) [file 12912_2018_291_MOESM1_ESM.docx]

**Additional File 1** Recruitment Protocols for undergraduate and nurse practitioner students

*Recruitment and randomization of undergraduate students*

A total of 393 third and fourth year undergraduate student nurses at the university and 58 third year student nurses at the college were identified as eligible for inclusion. Forty-three students responded and 22 agreed to participate; 21 were excluded due to no response, declined participation, and did not meet eligibility requirements. Once consented, 22 students were randomly assigned to either Group I or Group PI (note: one participant per group withdrew after randomization but before participating in study protocol); 20 undergraduate students completed all phases of the study according to their group assignment. The Research Assistant (RA) conducted a computerized randomization process to assign students to Group I or Group PI.

Recruitment strategies included in-class invitations by the lead researcher (ML) and the RA, booth advertisement at a student research day, email invitations, social media including Twitter, Instagram, and Facebook, posters, and written invitations left at the nursing reception desk. The advertisements in social media, emails, and poster stated: “We are interested in learning how to help student nurses discuss wellness and health risk behaviors with family carers.” Facebook garnered the most student responses at the college while email invitations (sent from the research office or nurse preceptor) generated the most student responses at the university.

*Recruitment and randomization of nurse practitioner students*

A total of 23 first-year nurse practitioner students enrolled in a clinical practice course at the university were eligible for inclusion; 22 consented to participate. A computerized randomization process was used by the RA to assign students to intervention groups. The sole recruitment strategy involved one in-class invitational session led by the lead researcher (ML) and the RA. Students who chose not to participate in the study were provided with an alternative activity that allowed them to earn course credits comparable to what they would earn were they to participate in the research project.
