# Supplementary material for: Heart health whispering: A randomized, controlled pilot study to promote nursing student perspective-taking on carers’ health risk behaviors
Source: BMC Nurs. 2018 May 24;17:21. doi: 10.1186/s12912-018-0291-1 (PMC5968556; doi:10.1186/s12912-018-0291-1)
Supplement: Supplementary file 2 — Intervention Protocol. (DOCX 19 kb) [file 12912_2018_291_MOESM2_ESM.docx]

**Additional File 2** Intervention Protocol

**Phase One (Group I only)**

*Phase One* was a one-time 20-minute instructional session conducted by the interventionist (JL) in-person or by telephone (student’s preference) about perspective-taking and challenges that students encounter when comprehending individuals’ motives for engagement in health risk behavior. The instructional session focused on imagine-other perspective-taking [1] by the student who was taught about: (a) identifying with his or her own thoughts and feelings about health risk behaviors, (b) putting aside his or her own thoughts or feelings to mentally ‘get inside the carer's shoes’ and discern the ‘root’ of the health risk behavior from the carer's viewpoint, and (c) sensitively communicating and validating his or her inferences with the carer [2].

The 20-minute instructional session was administered two weeks prior to the students’ scheduled session at the lab where the video-recorded dialogue with a carer about a health risk behavior took place. At the time of the instructional session, the RA provided each Group I (full intervention) student with an instructional sheet that defined thoughts and feelings, as well as outlined a guideline for practicing empathic dialogue. The instructional sheet directed students to practice perspective-taking over the next two weeks and focus on: (a) how they interpret a health risk behavior by a family member or a friend; (b) inferring what their family member or friend is thinking and/or feeling about the health risk behavior; and (c) having a discussion with the family member or a friend to validate if the student’s inferences are similar to the family member’s or friend’s viewpoint on the health risk behavior. About two days in advance of each student’s scheduled appointment at the lab, the RA sent a reminder email about the student’s upcoming lab session.

Group PI (partial intervention) students did not engage in the Phase One instructional and practice sessions. Instead, they were instructed to independently review communication skills previously learned in their coursework. In addition, the RA sent Group PI students an email reminder of their lab appointment typically two days in advance of the scheduled session. Along with the email reminder, a tip sheet that defined thoughts and feelings (but had no instruction for perspective-taking dialogue practice) was provided. Each Group PI student was given an opportunity to review the tip sheet once more at the lab prior to dialoguing with the carer in case the student did not have a chance to review the sheet before that point.

**Phase Two (Groups I and PI)**

*Phase Two* was administered two weeks later. Each student in Group I and Group PI

was individually paired with the blinded carer actor (aka the ‘carer’) to engage in a 10-minute video-recorded conversation about a health risk behavior identified by the carer. The carer arrived at the lab approximately 15 minutes after the student’s scheduled appointment in the lab. Once the caregiver actor arrived at the lab, the RA would greet the carer and instruct her to identify one health risk behavior to discuss with the student and report her ‘readiness to change’ score for that behavior. The carer then waited quietly in the observation room to begin the dialogue with the student. Carers in this study identified either smoking, binge-eating, poor diet, misuse of alcohol, or lack of exercise as the focus of the dialogue.

Upon arrival at the lab, the interventionist (JL) provided the student with: a brief written description about the carer they were to meet, the health risk behavior, the ‘readiness to change’ score identified by the carer, instructions to engage in a dialogue on the health risk behavior identified by the carer, and baseline measures (demographic and clinical empathy) to complete. Students were reminded that they were not to engage in an intervention with the carer and should instead focus on the precipitating factors (or triggers) underlying the carer’s health risk behavior. Group I students were asked to draw on their perspective-taking skills that they learned in Phase One. Group PI students were asked to draw on their previously learned communication and listening skills, and tip sheet. In total, Phase Two (instructions and video-recorded dialogue) took approximately 30 minutes to complete with the student and carer.

**Phase Three (Groups I and PI)**

*Phase Three* is based on a modification of Ickes’ [3] paradigm to capture students’ empathic accuracy. This phase consisted of the interventionist (JL) accompanying the carer to the video-feedback room to video-tag thoughts and feelings that the carer distinctly remembered having experienced, shared, displayed, or conveyed to the student during the dialogue. The carer indicated where she wanted to start and stop the video-file and record her tags in Studiocode™. Studiocode™ captured the time of each video-tagged instance, the self-reported ‘thought’ or ‘feeling’ experienced in the instance, the positive or negative tone of the tagged instance, and the situation in relation to the thought or feeling as expressed in a full sentence. The video-tagging exercise took the carer approximately 45 to 60 minutes to complete.

While the carer was engaged in the video-tagging exercise, the second RA (ML or LH) invited the student to quietly watch (no task required) the video-recorded conversation at a different computer in a private room. To make economical use of the student’s time in the lab, the student was then asked to complete the post-dialogue clinical empathy tool and respond to scripted open-ended exit interview questions about the study protocol up to that point (i.e., Group I students were asked questions about Phases One and Two; Group PI students about Phase Two; discussed further below).

After the carer completed the video-tagging exercise, the student immediately engaged in a similar video-tagging exercise that took 45 to 60 minutes to complete. Before the start of the exercise, the RA reviewed the video-tagging instructions with students as well as definitions for thoughts and feelings. Group I and PI students referred to these definitions as needed throughout the tagging process. For the video-tagging exercise, the interventionist showed students the dialogue video and paused it at each instance of the carer’s tags. At each instance, students were asked to infer: a) whether the carer experienced a thought or feeling, b) whether the tone of the tagged instance was positive or negative, and to describe c) the situation in relation to the carer’s thought or feeling which they expressed in a full sentence as if they were the carer (e.g., I am feeling embarrassed because of my smoking habit). The carer’s thoughts and feelings were not shared with the student.

**Phase Four (Groups I and PI)**

*Phase Four* involved conducting separate exit interviews with the carer and the student. While the student was engaged in the Phase Three video-tagging exercise, the second RA (ML or LH) asked the carer to complete two respective tools on the student’s clinical empathy and the carer’s readiness to change the health risk behavior, and respond to exit interview questions in a different room. Immediately after the student completed Phase Three, the interventionist (JL) asked the student to complete final exit interview questions about the video-tagging exercise and provide feedback on any improvements to the study protocol. The interviewers (ML or LH) and interventionist (JL) conducted open-ended exit interviews guided by an investigator-developed script (Tables 1 and 2) with each student and carer, separately. Hand-written notes captured participants’ evaluative responses about the impact, appropriateness, and acceptability of the empathic coaching session (Phase One; Group I only), the video-recorded dialogue (Phase Two), and the video-tagging session (Phase Three). Students and carers were prompted for suggestions to help the investigative team further refine the intervention. The exit interview questions with the students and carers, respectively took 30 minutes to complete.

In total, Phases One to Four with Group I students was completed in 140 minutes and Phases Two to Four with Group PI students was 120 minutes.

**References**

1. Batson CD, Early S, Salvarani G. Perspective-taking: imagining how another

feels versus imagining how you would feel. Pers Soc Psychol Bull. 1997;23:751-758.

2. Lobchuk M, Halas G, West C, Harder N, Tursunova Z, Ramraj C. Development of a novel

empathy-related videofeedback intervention to improve empathic accuracy of nursing

students: A pilot study. Nurse Educ Today. 2016;46:86-93.

3. Ickes W. Measuring empathic accuracy. In: Hall JA, Bernieri FJ, editors. Interpersonal

Sensitivity. New Jersey: Lawrence Erlbaum Associates, Inc.; 2001.
