# Supplementary material for: Heart health whispering: A randomized, controlled pilot study to promote nursing student perspective-taking on carers’ health risk behaviors
Source: BMC Nurs. 2018 May 24;17:21. doi: 10.1186/s12912-018-0291-1 (PMC5968556; doi:10.1186/s12912-018-0291-1)
Supplement: Supplementary file 3 — Family Carer Actor qualitative table. (DOCX 25 kb) [file 12912_2018_291_MOESM3_ESM.docx]

| **Additional file 3** Frequency Counts and Illustrative Extracts of Family Carer Actors’ Perceptions of Students’ Approach | | | | | | | | |
| --- | --- | --- | --- | --- | --- | --- | --- | --- |
| Theme | Subtheme | Example actor comments and counts by group | | | | | | |
|  |  | Partial intervention | *n* | | Full intervention | | *n* | |
| Perception of student’s behavior or approach |  |  |  | |  | |  | |
| Favorable | Good communication and listening skills | “I like how she stayed with me throughout the interview by listening intently.” (D9 UG) | 68 | “I felt truly listened to.” (D5 UG) | | 62 | |  |
|  | Nonverbal communication skills | “Her body language was congruent with her verbal comments and expressions.” (D9 NP) | 12 | “She used direct eye contact and didn’t move much, but conveyed listening.” (D18 NP) | | 12 | |  |
|  | Summarization | “She kept summarizing what she heard. She made a real effort this way and kept repeating what she heard me say.” (D9 NP) | 1 | “I liked the student’s summary of today’s dialogue.” (D5 UG) | | 8 | |  |
|  | Responsiveness | “She had a very caring, validating… approach.” (D3 UG) | 7 | “This student was intuitive in responding in ways that she inferred I needed.” (D5 UG) | | 8 | |  |
|  | Empathy | “Clearly [the student] had empathy.” D4 NP) | 5 | “[The student] was good at walking the line of non-judgment and being empathetic.” (D20 NP) | | 7 | |  |
|  | Gentle, educational approach | “She did not pressure me to change my smoking behaviour.” (D2 UG) | 10 | “She was gentle. She wasn’t talking down. She made me feel like I was on the right track without going over the top.” (D20 UG) | | 9 | |  |
|  | Liking the student or their approach | “I felt like I clicked with her.” (D21 NP)  “Because of her approach, she helped me to make that link by reigning me in to focus on my self-care and my smoking.” (D11 UG) | 37 | “I liked her.” (D19 NP)  “Overall this student was good. She asked the right questions.” (D14 NP) | | 45 | |  |
| Unfavorable | Paternalistic | “It seemed that he was advising me to do what works for him in his family.” (D8 UG) | 4 | “I think her approach was based on making a lot of assumptions and she spoke a lot for me. She was a bit patronizing.” (D16 NP) | | 21 | |  |
|  | No delving and/or jumping to intervention | “I don’t think she asked me a lot about the smoking … she didn’t delve into triggers leading to my smoking behaviour.” (D7 UG) | 9 | “I didn’t feel I had an opportunity to elaborate. She was too quick to jump to the next question. She appeared to be too eager and would cut me off.” (D13 NP) | | 21 | |  |
|  | Inauthentic | “She would look away after she asked me a question…brush away something from her clothing which felt odd to me…This behaviour suggested disinterest in my answers to her questions.” (D10 UG) | 1 | “I found myself wanting to talk to a ‘person’ (not a robot)... I could almost predict what she was going to say to me.” (D15 UG) | | 7 | |  |
|  | Student needed guidance | “I took responsibility for the conversation.” (D11 NP) | 14 | “I felt like I was leading the conversation a lot.” (D22 NP) | | 19 | |  |
|  | Student needs practice | “It will come with practice.” (D11 NP) | 3 | “She was trying hard I could tell by her body language but communication was limiting…I believe this student just needs more practice to feel more comfortable with taking a perspective-taking stance” (D4 UG)  “With experience, he’ll be fine.” (D17 UG) | | 6 | |  |
|  | Use of silence | “I also felt that the student was uncomfortable with several instances of silence during the 10-minute dialogue.” (D3 UG) | 5 | “When there were silences, they were awkward and painful, so I tried to avoid them by going on tangents.” (D17 UG) | | 3 | |  |
| Approach outcomes |  |  |  |  | |  | |  |
| Favorable | Felt understood | “Her paraphrasing tendency helped me to feel understood. I felt she got it right. I felt like I didn’t need to say more.” (D9 UG) | 25 | Overall this student’s approach made her feel “hopeful”, “validated”, and “understood.” (D5 UG) | | 27 | |  |
|  | Felt safe and comfortable in not being judged | “I felt that as the conversation progressed, I felt progressively more comfortable talking with her… she let me be open with her.” (D17 NP) | 44 | His questions made her feel comfortable enough to expose and explore things about herself. (D17 UG) | | 46 | |  |
|  | Felt new resolve to change | The actor thought that she would be able to discuss her stage of readiness to change at their next interaction. The actor reflected that “talking makes you take ownership.” (D23 NP) | 7 | “Hearing it from someone else helped me to consider change.” (D1 UG) | | 16 | |  |
|  | Learned to take the initiative | “I felt it is worthwhile being open about myself and my behaviour. It felt good to be understood and even relieved in being authentic. I will try this approach with other health care providers.” (D17 NP) | 9 | “I learned I sometimes need to take the initiative to share my context with my health care professionals and offer up more information about my situation” so they can help her overcome barriers to change behaviours. (D5 UG) | | 19 | |  |
|  | Learned about own feelings | The student’s approach “made me realize how resentful I was of the caregiving situation and feelings of guilt that I wasn’t aware of … otherwise I tended fight those feelings off without realizing how deep those feelings were.” (D7 UG) | 12 | “I learned that if I am not comfortable with someone, I will back off sharing information, even with my own physician.” (D15 UG) | | 13 | |  |
| Unfavorable | Not feeling heard | “I kept telling him that it can’t involve my family” and I wasn’t sure he was hearing this from me.” (D8 UG) | 3 | “I didn’t feel heard.” (D5 UG) | | 30 | |  |
|  | Uncomfortable | “I was still very forthcoming with information, but her approach made me feel awkward.” (D11 NP) | 7 | “I never felt that I relaxed into the conversation.” (D15 UG) | | 10 | |  |
| Mixed | Experienced mixed emotions | “I felt listened to and understood but then he was persistent about his advice to talk to my family more…” (D8 UG) | 4 | “I felt safe with her. On the other hand, I thought for a moment ‘is she really getting me?’” (D19 UG) | | 10 | |  |

UG = undergraduate student; NP = nurse practitioner student

Note: *n* captures multiple comments made by individual carer actors in the same sub-theme.
