# Supplementary material for: Heart health whispering: A randomized, controlled pilot study to promote nursing student perspective-taking on carers’ health risk behaviors
Source: BMC Nurs. 2018 May 24;17:21. doi: 10.1186/s12912-018-0291-1 (PMC5968556; doi:10.1186/s12912-018-0291-1)
Supplement: Supplementary file 4 — Student qualitative table. (DOCX 25 kb) [file 12912_2018_291_MOESM4_ESM.docx]

**Additional file 4** Frequency Counts and Illustrative Extracts of Students’ Perceptions of the Intervention and Their Approach

| Intervention phase | Theme | Subtheme | Example student comments and counts by group | | | |
| --- | --- | --- | --- | --- | --- | --- |
|  |  |  | Partial intervention | *n* | Full intervention | *n* |
| Phase 1: Instructional session and practice exercise | Consequences of practice session for self or other | Student’s better comprehension of other | N/A | N/A | “It [the approach] helped me to better understand his health risk behaviour.” (D22 NP) | 21 |
|  |  | Session stimulates thinking in other | N/A | N/A | The student thought the practice session made her mother think more about her behaviour. (D4 UG) | 8 |
|  | “What was practicing like” | Not easy to do | N/A | N/A | Engaging in perspective-taking was “hard to do.” (D1 UG) | 16 |
|  |  | Different | N/A | N/A | “I was a lot nicer to him than usual.” (D12 UG) | 9 |
| Phase 2: Video-recorded session | Communication techniques used to seek understanding |  | “I validated what she was trying to tell me.” (D9 UG) | 80 | “I also asked focused questions. I tried to maintain an open posture in the chair.” (D10 UG) | 56 |
|  |  | Perspective-taking | “I tried to come at understanding from the caregiver’s viewpoint” (D6 UG) | 16 | “I found myself trying to put myself in my mind in their shoes.” (D3 NP) | 23 |
|  |  | “Limited” training | “I don’t have formal communication training.” (D7 NP) | 5 | “Because we are not taught to listen and explore, I found myself biting my tongue to not intervene.” (D19 NP) | 4 |
|  | Approach outcomes for self | Sadness | “I felt sad for her and it’s what drew me into her situation.” (D9 NP) | 2 | “I felt sadness toward Donna and in hearing her caregiving situation.” (D3 NP) | 5 |
|  |  | Gained appreciation of the carer’s perspective | “Drinking helped to relax her which I understood.” (D12 NP) | 28 | “It [the empathic technique] helped me to see how people cope and understand their unique reasons to help them cope.” (D19 UG)  “The technique helped me to learn about smoking habit deeper. I never delved this deeply with ‘real’ patients I encountered in my clinical experiences.” (D15 UG) | 41 |
|  |  | Self-reflection on improvement | “I feel that I have lots to learn yet about communication and it will come with practice!” (D8 UG)  “I feel that I was all over the place” (D6 UG) | 40 | “I should have used silence more” (D14 NP) | 27 |
|  | “What was it like” | Self-awareness | “I was very self-aware of myself.” (D12 NP) | 17 | The student explained that she became more cognizant of her own body cues (e.g., nodding) mid-way into the dialogue (D5 UG) | 24 |
|  |  | A positive experience | “I did feel more comfortable over time” (D21 NP) | 15 | “It felt good.” (D19 NP) | 12 |
|  |  | Wanted to intervene | “As a health care provider, you want to provide.” (D4 NP) | 13 | “I felt useless with just listening.” (D4 UG) | 18 |
|  |  | Not easy to do | “It’s hard to ask tough questions” (D11 NP) | 14 | “Initially it was awkward because I felt delving into one’s personal circumstances can cause embarrassment. It’s like addressing the big elephant in the room.” (D15 UG) | 13 |
|  |  | Different | The student explained that the interaction is different with only chance opportunities to dialogue whenever she had tasks at the bedside. The interaction today with Donna was more focused, with more time to talk about her concerns. (D7 NP) | 7 | “Normally I interrupt people a lot to get on with what I need to do. I don’t mean to but I realize that I can get more information when I am quiet with people.” The student went on to explain that this was a different experience where she was specifically asked to not intervene as that’s what nurses are trained to do. (D19 NP) | 10 |
|  |  | Felt nervous | The student said that the experience was “nerve-wracking. The nerves were there.” (D10 NP) | 7 | The student said that she was “nervous.” (D3 NP) | 3 |
| Phase 3: Video-Tagging | Appreciation |  | The student stated she thought reviewing the discussion with the caregiver was a good way to learn “what questions or prompts to ask” or “what topics to focus on.” (D10 UG) | 10 | The student called watching her dialogue “eye-opening.” (D12 UG) | 17 |
|  | Self-reflection on improvement |  | After watching the video, the student thought that maybe he wasn’t pro-active enough. He allowed the caregiver actor to talk about her feelings, but could have looked at what was driving those feelings. “I think I missed the boat on that.” (D10 NP) | 11 | “I can’t believe how much I missed in the conversation … I should have validated Donna’s feelings more. I thought I was doing that, but I see that I wasn’t really!” (D12 UG) | 15 |

UG = undergraduate student; NP = nurse practitioner student

Note: *n* captures multiple comments made by individual students in the same sub-theme.
